# Editorial: Advances in the neurobiology and neuropsychology of offending behaviour

**DOI:** 10.3389/fpsyt.2026.1777538

**Published:** 2026-01-20

**Authors:** Najat R. Khalifa, Birgit A. Völlm

**Affiliations:** 1 Department of Psychiatry, Queen’s University, Kingston, ON, Canada; 2 Klinik und Poliklinik für Forensische Psychiatrie, Universitätsmedizin Rostock, Rostock, Germany

**Keywords:** brain stimulation, facial recognition, mental health, neurobiology, neuropsychology, offending

Research shows that offending behaviour is influenced by a complex interplay of biological, developmental, psychosocial, and environmental factors (1–4). For many decades, the quest to elucidate the neurobiological and neuropsychological underpinnings of offending behaviour has been the subject of scientific enquiry, particularly in areas such as genetics, neuroimaging, psychophysiology, neurochemistry, cognitive neuroscience, behavioural neuroscience, and others. Although the current body of scientific knowledge has significantly advanced our understanding of the neurobiological and neuropsychological antecedents of offending behaviour, research in this field is continuously evolving.

Therefore, this Research Topic of the Forensic Section of Frontiers in Psychiatry aims to bring together an up-to-date Research Topic of scholarly work in the field, providing academics, practitioners, and policymakers with information about the latest advances in the neurobiology and neuropsychology of offending behaviour. The scope of this Research Topic is broad, and includes scholarly work pertaining to psychophysiology, facial and emotional recognition, non-invasive brain stimulation, traumatic brain injury, treatment outcomes, and other areas.

Using data from a longitudinal twin project involving 1,082 children from Los Angeles (USA) who underwent successive waves of testing, Bertoldi et al. examined skin conductance (SC) activation during anticipation of and in response to a noise stressor in a sample of 9–10 year old children in relation to constituent traits of psychopathy as per the triarchic model (e.g., boldness, meanness, disinhibition), both concurrently and at a five-year follow-up, as well as antisocial behavior at the follow-up. The findings revealed reduced SC reactivity in the noise-stressor task at wave 1 (ages 9–10 years) was related to parent-rated boldness both concurrently and prospectively at wave 3 (ages 14–15 years), but not to parent-rated disinhibition or meanness at either wave. Additionally, reduced SC reactivity at wave 1 was associated with both boldness and meanness as well as nonaggressive antisocial behaviour at wave 3 as rated by child participants, whereas parent-rated boldness at wave 1 was predictive of child-reported aggressive behaviour at wave 3. These findings highlight the intricate relationship between low fear (threat sensitivity) and the construct of psychopathy, in particular fearlessness.

Hachtel et al. examined differences in subjective and neuroendocrine responses to psychosocial stress and their impact on facial emotion recognition (FER) and performance on an arithmetic task in justice-involved individuals with schizophrenia and healthy controls. The results showed significant group differences in FER, and a significant increase in the subjective perception of momentary strain relating to psychosocial stress in both groups. Notably, acute psychosocial stress was reported to enhance FER performance in a sub-task related to naming emotions in the patient group. In a separate study, Tiberi et al. examined the recognition of dynamic facial expressions of emotion in 112 male participants: forensic inpatients with a history of sexual offenses, forensic inpatients with a history of non-sexual offenses, and community controls, using the Signal Detection Theory indices of sensitivity and response bias, as well as measures related to reaction time, emotion labeling reflection time, task easiness, and easiness reflection time. The results showed that both forensic groups were more likely than the control group to exhibit emotion recognition deficits. Except for surprise which was selected less by those with a history of sexual offending, deficits were found in all emotions rather than – as in previous studies – for particular ones, such as anger or disgust. Interestingly, despite poorer performance, both forensic groups rated the task as easier compared to the control group. Together, the findings of these studies highlight the importance of developing therapeutic interventions aimed at enhancing stress resilience and emotion recognition in justice-involved individuals.

Traumatic brain injury featured in two contributions to this Research Topic. In a cross-sectional study involving a sample of 286 adult male prisoners in Scotland, McMillan et al. found severe head injury (SHI) in 245/286 (86%) and repeated head injury in 151/245 (62%). They also found that SHI was significantly associated with disability, problematic drug or alcohol use, clinical anxiety, and clinical depression, but not cognitive test outcomes. Additionally, those with SHI had higher rates of arrests, charges, and convictions and at younger ages, as well as violent and property offences than those without SHI. In a separate study in New Zealand, Theadom et al. used a case control research design involving a large sample of 6,606 individuals with mild traumatic brain injury (mTBI) and 15,771 matched trauma controls. The results revealed that 10 years post injury, people experiencing a single mTBI had significantly higher numbers of violent charges and convictions but not other, non-violent charges and convictions. Subgroup analysis showed that those with a history of prior mTBIs had significantly higher numbers of violent charges and violent convictions. For those having experienced multiple mTBI the link with number of violent criminal convictions and charges was stronger, suggesting a cumulative effect. Importantly, these findings were only significant for men, not for women. Together, the findings of these studies highlight the association between traumatic brain injury and many undesirable outcomes including disability, psychopathology, and offending behaviours, especially violence.

Furthermore, a case report by Karcher et al. involving a young man with behavioural variant of frontotemporal dementia, signifies the impact of brain psychopathology on behavioural changes and the challenges inherent in conducting forensic evaluations in people with major neurocognitive disorder of frontotemporal type.

Impulsivity, a tendency to act rashly and without forethought, and its correlates were the subject of two studies in this Research Topic. Hoffmann and Völlm examined the association between neuropsychological deficits, in areas of attention, executive function, and social-emotional cognition, and impulsivity and criminal history in a sample of 30 male patients with substance use disorders at a forensic psychiatry clinic in Germany. The results showed that individuals with substance use disorders displayed significantly higher levels of impulsivity, as measured using the Barratt Impulsiveness Scale (BIS-11), than the general population. They also showed significant deficits in attention, psychomotor speed, and executive functions as measured using the Cambridge Neuropsychological Test Automated Battery (CANTAB). The results also showed that although there was a modest correlation between impulsivity and cognitive performance, extensive criminal histories correlated with poorer cognitive performance, particularly in tasks requiring planning and problem-solving. These findings highlight the nuances inherent in the relationship between cognitive deficits, impulsivity, and criminal history, and the need for tailored assessments and rehabilitation strategies to enhance outcomes for justice-involved people with substance use disorder.

Khalifa et al. reported on the findings of a systematic review and meta-analysis to synthesise the literature on the use of combined cognitive training and NIBS to modulate impulsivity and its subdomains (motor, delay discounting, reflection). A synthesis of findings from four randomised controlled studies involving the use of combined cognitive training and tDCS in 127 subjects with substance use disorders, obesity, and Parkinson’s disease revealed that combined cognitive training and tDCS had no statistically significant effects on motor impulsivity as measured using the Stop Signal Task, Go/No Go, and a delay discounting task. The authors noted that there is a dearth of literature on the use of combined cognitive training and NIBS for impulsivity, highlighting the need for more research in the field.

Finally, we hope that this Research Topic of scholarly work provides new insights into some neurobiological and neuropsychological aspects of offending behaviour that may help identify targets for prevention and intervention as well as areas for future research in the field.
